# Reply to “Methodological caveats in interpreting slow-wave slope as a marker of synaptic homeostasis in OSA”

**DOI:** 10.1007/s44470-026-00203-7

**Published:** 2026-10-13

**Authors:** Rama Maganti, Jun Wang, Scott Hetzel

**Affiliations:** 1 Department of Neurology, School of Medicine and Public Health, University of Wisconsin, 1685 Highland Ave, Madison, WI 53706 USA; 2 Department of Biostatistics and Medical Informatics, School of Medicine and Public Health, University of Wisconsin, Madison, WI USA

We thank Drs. Huang and Shen for their thoughtful comments [1] regarding the interpretation of slow-wave slope in our study of “Sleep homeostasis impairment across obstructive sleep apnea severity: findings from a population-based cohort study” [2]. We agree that scalp EEG-derived slow-wave slope is not an exclusive marker of synaptic dynamics and that physiological and structural factors can influence its measurement. Widespread structural brain abnormalities, including white matter injury and cortical changes, were shown in OSA that may alter neuronal synchronization and signal propagation [3]. Furthermore, any change caused by a structural brain abnormality would likely be persistent throughout the night and, in part, may contribute to changes in scalp-recorded slow-wave characteristics. We acknowledge the lack of imaging as a limitation of our study.

We respectfully disagree that the absence of an overnight decline in slow-wave slope among individuals with severe OSA is attributable to a floor effect. Table 1 of the manuscript provided the group means and SDs. The means ranged from 214.2 to 219.3 while the SDs were fairly consistent around 25.0. A possible floor effect would require many participants (> 15%) [4] to be near the threshold of 0. The minimum values across the groups ranged from 130 to 165. The patients in this study did not experience a floor effect to limit possible differences in slow-wave slope. Thus, the lack of an overnight decline in slow-wave slope is more consistent with impaired sleep homeostasis.

As to mathematical coupling, the slow-wave decay slope was estimated by regressing hourly mean SWA over time for each participant. Since the slope of the *mean* SWA measures over time is not directly linked to the slope of SWA measures specifically during the first hour of NREM sleep, then these two variables are not mathematically coupled.

We also note that the interpretation of scalp EEG-derived slow-wave slope as a marker of synaptic dynamics and sleep homeostasis is supported by experimental evidence. A seminal high-density EEG study showed that slow-wave slope is tightly linked to sleep homeostatic pressure, decreasing across the night with the dissipation of sleep need [5]. Likewise, using cortical recordings and local field potentials, another study showed that slow-wave characteristics, including slope, track sleep–wake history and reflect underlying changes in synaptic strength associated with wake-dependent potentiation and sleep-dependent downscaling [6]. Together, these studies provide direct electrophysiological evidence supporting slow-wave slope as an indicator of synaptic homeostasis.

Finally, our conclusions were not based solely on slow-wave slope. We observed convergent abnormalities across multiple independent measures of sleep homeostasis, including attenuated overnight decay of slow-wave activity and altered relationships between wake after sleep onset and subsequent slow-wave activity [2]. The consistency of these findings across complementary homeostatic measures argues against a single methodological explanation and supports the broader conclusion that severe OSA is associated with impaired sleep homeostasis.

We appreciate Drs. Huang and Shen’s insightful comments and fully agree that future studies combining polysomnography, advanced neuroimaging, and multimodal neurophysiological biomarkers will be important for disentangling the relative contributions of structural brain alterations and synaptic dysregulation to abnormalities in sleep homeostasis observed in OSA.

## Data Availability

All polysomnography data from the Wisconsin Sleep Cohort are available as part of the National Sleep Research Resource and can be directly obtained by communicating (support@sleepdata.org). The Python and MATLAB scripts used for analysis will be made available upon reasonable request to the corresponding author.
